# Physical child abuse and self-reported health concerns: A case-control study including police-reported cases and unreported controls

**DOI:** 10.1371/journal.pone.0330601

**Published:** 2025-09-02

**Authors:** Daniella Justesen, Carl Johan Wingren, Liselott Slot, Maria Balsløv, Andrea Lykke Thanning, Jytte Banner

**Affiliations:** Department of Forensic Medicine, Section of Forensic Pathology, University of Copenhagen, Copenhagen, Denmark; Neighborhood Physical Therapy, UNITED STATES OF AMERICA

## Abstract

**Background:**

Child abuse continues to pose a significant threat to children’s health. The repercussions of abuse are profound, impacting the child’s physical, social, and emotional well-being, with potential long-term effects that may extend into adulthood. To assist in identifying health concerns in children associated with exposure to physical abuse, a health questionnaire was developed to be used in the setting of a forensic examination.

**Objective:**

This study examines whether children suspected of being exposed to physical violence report more health-related concerns compared to unexposed controls.

**Participants and setting:**

The case group consists of children suspected of being exposed to physical violence, with reports to the Copenhagen police. Cases were examined from April 1, 2020, to December 31, 2023, at the Child Advocacy Centre (CAC) in Copenhagen, totaling 374 examinations. A control group of children aged 4–14 years with no suspicion of abuse was established through recruitment via social media platforms (Facebook, LinkedIn), posters, and word of mouth. Controls were examined from November 1, 2023, to September 30, 2024, totaling 122 examinations.

**Methods:**

Children underwent a standardized forensic examination, which included a health interview reviewing health behaviors (e.g., diet, toothbrushing, and sleep patterns) and well-being (liking school/preschool, having friends, and trusted adults).

**Results:**

Overall, cases reported significantly more concerns than controls on several assessed items. With multivariate logistic regression, adjusted for all significant covariates and stratified by age, two concerns remained significant. Cases aged 8–14 years, had significantly higher odds of brushing their teeth once daily or less (OR: 3.85; CI: 1.47–10.12) and reported low enjoyment of school (OR: 3.74; CI: 1.03–13.53).

**Conclusions:**

Health interviews may support the identification of children at risk. However, the statistical power was limited, and the findings require validation in larger populations.

## Introduction

Every year, approximately 55 million children in Europe are affected by various forms of violence [1]. Considering non-reported cases, 208 million children are affected, with 9.6% experiencing sexual abuse, 22.9% experiencing physical abuse, and 29.1% experiencing emotional abuse [1]. Recently, a national self-reported survey of Danish 8th grade children indicated that 22% reported experiencing physical abuse and 23% reported emotional abuse [2], consistent with estimated European prevalences.

The long-term consequences associated with violence against children are extensive, including physical, mental, and emotional impairment [3–11]. Psychosomatic symptoms, somatic complaints, overweight, lack of toothbrushing, sleep disturbances, bullying, and school disengagement have all been associated with child maltreatment [6,7,12–17].

Due to the complexity of child abuse, many available identification tools lack validity and are low on specificity and sensitivity [18–21]. Among the available tools, many are retrospective questionnaires, interviews, or physical examinations, primarily designed for emergency departments and trauma centers, e.g., TEN-4-FACEp and SPUTOVAMO-R [18,19,21,22]. Of the questionnaires, the most studied and validated tool is the Childhood Trauma Questionnaire (CTQ), a 28-item questionnaire that covers physical, sexual, and emotional abuse and physical and emotional neglect [21]. However, few tools are designed to identify child neglect [20].

The Children’s Somatic Symptoms Inventory, developed by Walker et al., has been widely used in clinical settings to measure somatic distress [23–25]. The revised version of the inventory, validated for use with children aged eight years and older, comprises eight questions addressing nausea, dizziness, fatigue, headache, abdominal pain, back pain, and pain in arms and legs [24]. These symptoms have also been linked to child abuse [6,7,26]. The health and well-being of school-aged children (11–15 years) are monitored through national and international surveys every four years [27,28], as well as through regular examinations by the general practitioner and health visitors. In Denmark, health visitors typically conduct health interviews with children, around 1st grade, 5th and 8th grades of elementary school [29]. These interviews are guided by the Danish Health Authority’s recommendations, which cover aspects such as the child’s well-being, diet, sleep, exercise, hobbies, vision, height, and weight [30].

While the long-term consequences of child maltreatment are well-documented, there is comparatively limited research on its short-term consequences, particularly regarding physical health and well-being in children. To our knowledge, only a few studies have addressed physical health complaints in children and adolescents exposed to abuse [6,26,31].

This study examines health and well-being characteristics of children with police-reported suspicions of physical violence, compared to non-exposed controls. Furthermore, it explores whether questions used during forensic examinations in Copenhagen, inspired by health visitors’ school interviews, could serve to identify early predictors of abuse in forensic and other settings.

## Materials and methods

### Case group

Data originate from a collaborative intervention project between the Section of Child Abuse at The Copenhagen Police, the Child Advocacy Center (CAC) in Copenhagen (i.e., Danish Children’s Centre), and the Department of Forensic Medicine in Copenhagen [32]. The included children were police-reported cases, suspected of being exposed to physical abuse perpetrated by their parents or family members. The children underwent forensic and dental examinations, including health-related interviews at the CAC Copenhagen, immediately following a video-recorded interview by the police. A total of 374 examinations were conducted, spanning from April 1, 2020, to December 31, 2023.

This study included children aged 4 to 14 years. If the child underwent multiple examinations, only the most recent assessment was included. Children were excluded if they were unable to cooperate due to fatigue or were unable to answer health-related questions. Additionally, children were excluded if participation in a health interview was not recommended by the police due to ongoing police investigations. A total of 352 children were included.

In Denmark, forensic interviews do not focus on disclosure or information about the violent incident, as in some other countries [33]. The investigation of violent incidents is conducted solely by trained police investigators and assessed through video-recorded interviews.

### Control group

A control group was established by recruiting families with children aged 4–14 years through social media platforms (Facebook, LinkedIn), a school in a nearby municipality to Copenhagen (utilizing the school’s communication platform), posters, and by word of mouth. The school “Pilehaveskolen” in the municipality of “Vallensbæk” has geographical similarities to the catchment area of the Copenhagen Police Department. Both municipalities are located in the Capital Region of Denmark, with “Vallensbæk” being a suburban area close to Copenhagen. A total of 122 controls were examined from November 1, 2023, to September 30, 2024.

### Forensic medical examination

Cases and controls underwent the same standardized examination, which included a health-related interview performed by a nurse and a top-to-toe body examination performed by a forensic doctor. When a dentist was available, children in the case group also received a dental examination. The health-related interview covered questions regarding diet, health-related behaviors (e.g., toothbrushing, physical activity), bodily functions (e.g., defecation, urination, sleep patterns), somatic distress (e.g., nausea, dizziness, pain), and whether the child enjoyed attending school or preschool. Questions regarding diet, health-related behaviors, physical functions, and enjoyment of school were adapted from the Danish Health Authority’s guidelines for Health Visitors conducting interviews with children. Additionally, the Children’s Somatic Somatization Inventory (CSSI-8) was used to assess somatic distress [24]. The interviews also included questions regarding the child’s history of medication and/or diseases/allergies, whether the child had followed the Danish childhood vaccination program and known difficulties or significant developmental delays during childhood. The top-to-toe examination documented all visible lesions on the skin of the body, head, and mouth. Examinations were standardized to ensure consistency in data collection and minimize potential variability.

The health-related interviews were conducted in two versions. Between April 1 and December 16, 2020, 99 children were interviewed using version 1. Between December 17, 2020, and December 31, 2023, a total of 253 children were interviewed using version 2, an updated and expanded version.

### Informed consent and participation procedure

For the case group, the police obtained consent from the children’s caregivers to conduct a forensic examination. On the day of the examination, consent was obtained from the child to participate in a forensic examination.

For the control group, the following procedure was implemented, as requested by the Regional Ethics Committee: 1) An informational meeting with the examining nurse and/or forensic doctor was conducted at least 24 hours before the examination. This meeting included the child and their family, ensuring they had sufficient time to consider their decision to participate.

2) On the day of the examination, informed verbal consent was obtained from the children and their parents in the presence of at least four persons (the child, one family member, the examining nurse, and the examining medical doctor) and 3) a written consent was obtained from the parents among controls on the day of the examination. If only one parent was present at the examination, and the parents had shared custody of the child, the parent was required to bring a power of attorney from the other parent authorizing the researcher to examine the child.

As a comforting procedure, but not as a request from the ethical committee, the children were informed that it was acceptable to respond with “I don’t know,” “I don’t want to answer,” or “I don’t want to be examined” without any consequences.

The nurse adapted and explained the meaning of the questions to the child during the health-related interview to enhance clarity and understanding. The responses were marked as missing if the child could not comprehend the question despite the nurse’s explanations. When answers did not fit into predefined categories, the nurse recorded the child’s responses as open-text entries or sought to reach a consensus with the child.

A checklist of the health questions assessed in the interview is available in Supporting Information S1.

### Ethical approval

The study was approved by the Danish Data Protection Agency through the University of Copenhagen [case numbers 514–0669/21–3000 and 514–0948/24–3000]. According to Danish legislation, no ethical approvals were required for the case group, as it is a registry-based study utilizing forensic routine data [case number 004–0022/18–7000] compared to registry data. The Danish Research Ethics Committee, Capital Region [journal number H-22055558] approved the use of the control group participants based on informed consent for the research described in this project. The project follows the Danish rules and principles for research and the Declaration of Helsinki [34].

### Description of study variables

The interview focused on various aspects of the child’s health and well-being, including diet (breakfast, lunch, dinner, sweets), sugary drinks (e.g., soda, ice-tea), health behaviors (toothbrushing, physical exercise), bodily functions (urination, defecation, sleep patterns), bodily distress (physical symptoms and pain), and school well-being (friends, likes school, trusted adults).

Irregular or rarely consumed breakfast, lunch, or dinner was considered concerning. Consumption of sweets and sugary drinks was considered concerning, with intake>=4 times per week. Irregular toothbrushing (once a day or less) and insufficient physical activity (less than one hour a day) were likewise considered concerning.

Bodily functions included daytime and nighttime wetting. Frequent daytime wetting was considered concerning for all ages, and nighttime wetting was considered concerning when frequent at ages 6 years and older, since younger children more often struggle with bladder control [35]. Additional concerns included frequent constipation or diarrhea, sleep less than < 8 hours per night [36,37], or two of the following: having frequent awakenings, nightmares, difficulty falling asleep, or not feeling rested in the mornings.

Bodily distress was evaluated using the revised Children’s Somatic Symptom Inventory (CSSI-8), [24,25]. Questions were adapted for all ages, and explanations were provided for specific symptoms to enhance understanding. Symptoms occurring weekly or daily were considered concerning.

Predictors of thriving and well-being in school or preschool were assessed by asking the child whether they liked attending school and whether they had friends and trusted adults. Negative responses to these questions were considered concerning.

A risk score was calculated by summing up the total number of concerns from the questions asked in the interview. This led to a low-risk group (0–2 concerns), a moderate-risk group (3–5 concerns), and a high-risk group (≥ 6 concerns), (Table 1). The cut-offs were determined by the inherent distribution of the data, creating three equally sized tertile groups.

**Table 1:** Risk group based on the number of concerns.

Number of concerns	Risk group
0-2	Low risk
3-5	Moderate risk
6-23	High risk

The total number of possible concerns was 23. Groups were compared based on their scores to evaluate whether cases had more concerns than controls and whether the number of concerns differed between genders.

A full description of the variables and how the interview was performed is available in Supporting Information S2.

### Statistical analysis

All analyses were considered statistically significant with *p*-value < 0.05 at the 95% confidence interval (CI). Age was not normally distributed and is reported as median and interquartile range. To investigate which items (predictors) of the questionnaire were correlated with outcome (case/control), non-parametric bivariate correlation analysis were performed, followed by univariate regression analyses. Significant predictors were included in multivariate logistic regression analyses. Analyses were performed unstratified and stratified by age, rather than including age as a covariate, since age was considered a key factor influencing how children interpreted the questions. We did not have access to sociodemographic data or other covariates of interest and were therefore unable to adjust our models with factors other than age and sex. Nagelkerke’s R^2^ was used to evaluate the usefulness of the model. Data were analyzed using Statistical Package for the Social Sciences (SPSS), version 29.0.1.0, 2022.

## Results

Data from 352 cases and 122 controls were analyzed. The median age was 8 years (IQR, 6–11 years) among cases and 7 years (IQR, 6–10 years) among controls. However, the proportion of older children was significantly higher among cases (*p*-value = 0,02).

Overall, the proportion of boys was similar among cases (178;51%) and controls (69;56,6%). However, when stratified by age groups, the distribution of girls and boys was approximately 60/40 in the youngest age groups, with girls being slightly overrepresented among the older children in both cases and controls (Table 2).

**Table 2:** Distribution of age and sex, stratified by age groups.

	Cases				Control			
Age	4-7 years		8-14 years		4-7 years		8-14 years	
Value	N (%)	Median	N (%)	Median	N (%)	Median	N (%)	Median
Male	84 (61.3)	5.98	94 (43.7)	9.82	42 (66.7)	5.55	27 (45.7)	9.86
Female	53 (38.7)	5.91	121(56.3)	10.75	21 (33.3)	5.69	32 (54.2)	9.93
Total	137		215		63		59	

Median is presented as group median.

Cases reported more concerns across nearly all variables assessed in the questionnaire (Table 3).

**Table 3:** Descriptives of study variables in cases and controls.

	Cases (N = 352)		Controls (N = 122)	
Variable of interest	Concern N(%)	Missing N(%)	Concern N(%)	Missing N(%)
Breakfast	70 (19.9)	< 5*	9 (7.4)	< 5*
Lunch	< 5*	< 5*	0	0
Dinner	6 (1.6)	10 (2.8)	0	0
Sweets	60 (17.0)	78 (22.2)	25 (20.5)	0
Sugary drinks	68 (19.3)	101 (28.7)	19 (15.6)	0
Toothbrush	85 (24.1)	36 (10.2)	11 (9.0)	0
Toothbrushing, assistance**	71 (28.3)	47 (18.7)	18 (18.2)	0
Exercise/physical activity	19 (5.4)	126 (35.8)	0	0
Daytime wetting	9 (2.5)	21 (6.0)	7 (5.7)	0
Nighttime wetting***	21 (6.0)	13 (3.7)	< 5*	0
Constipation/diarrhea	36 (10.2)	103 (29.3)	14 (11.5)	6 (4.9)
Sleep pattern	32 (9.1)	41 (11.6)	0	0
Nausea	25 (7.1)	156 (44.3)	< 5*	12 (9.8)
Dizziness	27 (7.7)	159 (45.2)	< 5*	10 (8.2)
Palpitations	17 (4.8)	161 (45.7)	< 5*	11 (9.0)
Fatigue	11 (3.1)	176 (50.0)	< 5*	12 (9.8)
Headache	39 (11.1)	35 (9.9)	8 (6.6)	13 (10.7)
Abdominal pain	29 (8.2)	40 (11.4)	5 (4.1)	14 (11.5)
Back pain	15 (4.3)	56 (15.9)	< 5*	23 (18.9)
Pain in arms/legs	18 (5.1)	50 (14.2)	7 (5.7)	15 (12.3)
School/preschool	41 (11.6)	101 (28.7)	5 (4.1)	< 5*
Friends	10 (2.8)	99 (28.1)	< 5*	< 5*
Trusted adults	14 (4.0)	129 (36.6)	< 5*	< 5*

* Exact numbers not shown to ensure anonymity.

**Includes only children < 11 years of age (n = 251, n = 99).

*** Includes only children > 5 years of age (n = 291, n = 94).

Missing data was more prevalent among cases, particularly in responses to some variables assessing somatic distress (CSSI-8). Results from univariate regression analyses showed a significant association between outcome (being in case group) and skipping breakfast (OR: 3.12; CI: 1.5–6.5), having a higher consumption of sugary drinks (OR 2.01; CI: 1.15–3.54), brushing the teeth once a day or less (OR 3.71; CI: 1.91–7.24), no assistance for toothbrushing if the child was 10 years or younger (OR 2.40; CI 1.33–4.32), having frequent nausea (OR 5.21; CI: 1.54–17.69), dizziness (OR: 8.95; CI: 2.09–38.38) or palpitation (OR: 5.33; CI: 1.21–23.50), disliking school/preschool (OR: 4.49; CI 1.73–11.68) and lacking trusted adults (OR: 7.90; CI: 1.03–60.87), (Table 4).

**Table 4:** Univariate regression analysis of variables.

Variable of interest	OR (95% CI)	*p*-value	Nagelkerke R^2^
Breakfast	3.12 (1.51-6.47)	< 0.01	3.6%
Sweets	1.09 (0.64-1.84)	0.75	0%
Sugary drinks	2.01 (1.15-3.54)	0.02	2.4%
Toothbrush	3.71 (1.91-7.24)	< 0.001	6.0%
Toothbrushing, assistance*	2.40 (1.33-4.32)	< 0.01	4.5%
Daytime wetting	0.46 (0.17-1.26)	0.13	0.7%
Nighttime wetting**	7.23 (0.96-54.52)	0.06	2.6%
Sleep pattern	0.83 (0.52-1.33)	0.44	0.2%
Constipation/diarrhea	1.23 (0.64-2.39)	0.54	0.1%
Nausea	5.21 (1.54-17.69)	< 0.01	4.5%
Dizziness	8.95 (2.09-38.38)	< 0.01	6.7%
Palpitations	5.33 (1.21-23.50)	0.03	3.2%
Fatigue	3.60 (0.78-16.56)	0.10	1.6%
Headache	1.77 (0.80-3.92)	0.16	0.8%
Abdominal pain	2.11 (0.80-5.60)	0.13	0.9%
Back pain	2.59 (0.58-11.53)	0.21	0.7%
Pain in arms/legs	0.91 (0.37-2.23)	0.83	0%
School/preschool	4.49 (1.73-11.68)	< 0.01	4.8%
Friends	4.94 (0.63-39.03)	0.13	1.3%
Trusted adults	7.90 (1.03-60.87)	0.05	2.8%

*Includes only children < 11 years of age, (n = 251. n = 99).

**Includes only children > 5 years of age (n = 291. n = 94).

Items without the possibility of being compared with the control group (zero observations in the control group) are excluded.

Since the age of the child could interact with the understanding of the questions and the interpretation of the concerns, we performed age-stratified analyses (groups < 8 years and >= 8 years). In these age-stratified analyses, the associations between concerns and health-related behaviors were similar to those observed in the unstratified analyses. However, skipping breakfast (OR: 7.82; CI 1.0–60.5) and having a higher consumption of sugared drinks (OR:3.15; CI: 1.26–7.87) were only significant among the youngest children, while the lack of trusted adults was not significant in either group (Table 5). Regarding somatic distress in children equal or older than 8 years, the effect size was reduced for frequent nausea (OR: 3.48; CI: 1.01–12.06) and dizziness (OR: 6.07; CI: 1.39–26.47) and became borderline significant regarding palpitations (OR: 7.53; CI: 0.98–57.91; *p*-value 0.053), (Table 5).

**Table 5:** Univariate logistic regression of variables, stratified by age.

Variable	4-7 years		8-14 years	
of interest	OR (95% CI)	*p*-value	OR (95% CI)	*p*-value
Breakfast	7.82 (1.0-60.5)	0.05	2.15 (0.96-4.81)	0.06
Sweets	1.79 (0.73-4.35)	0.20	0.72 (0.37-1.40)	0.34
Sugary drinks	3.15 (1.26-7.87)	0.01	1.41 (0.69-2.91)	0.35
Toothbrush	3.82 (1.39-10.48)	0.009	3.45 (1.41-8.47)	<0.01
Toothbrushing, assistance*	5.25 (1.94-14.29)	0.001	1.12 (0.51-2.50)	0.75
Daytime wetting	0.59 (0.19-1.85)	0.37	0.55 (0.05-6.20)	0.63
Enuresis**	5.42 (0.67-43.73)	0.11	*NA*	*NA*
Constipation/diarrhea	1.41 (0.56-3.54)	0.47	1.22 (0.47-3.21)	0.69
Nausea	*NA*	*NA*	3.48 (1.01-12.06)	0.05
Dizziness	*NA*	*NA*	6.07 (1.39-26.47)	0.02
Palpitations	*NA*	*NA*	7.53 (0.98-57.91)	0.05
Fatigue	*NA*	*NA*	5.02 (0.63-39.83)	0.13
Headache	1.50 (0.15-14.75)	0.73	1.39 (0.58-3.34)	0.46
Abdominal pain	1.19 (0.30-4.78)	0.81	3.02 (0.69-13.29)	0.14
Back pain	0.47 (0.03-7.78)	0.60	3.74 (0.48-29.16)	0.21
Pain in arms/legs	0.32 (0.05-1.98)	0.22	1.10 (0.35-3.44)	0.87
School/preschool	5.10 (1.12-23.29)	0.04	3.91 (1.14-13.41)	0.03
Friends	4.02 (0.47-34.24)	0.20	*NA*	*NA*
Trusted adults	4.47 (0.51-39.37)	0.18	*NA*	*NA*

* Includes only children < 11 years of age (n = 251. n = 99).

**Includes only children > 5 years of age (n = 291. n = 94){Citation}.

*NA* = analyses not possible due to too few cases.

While the univariate regression analyses revealed associations between several items and physical child abuse, these associations were reduced to only include toothbrushing (OR: 2.89–3.08; CI: 1.33–6.76) and dislike school or preschool (OR: 3.56–3.68; OR: 1.26–10.40) when performing a multivariate regression analysis and adjusting for gender (Table 6).

**Table 6:** Multivariate logistic regression of significant variables.

Variable of interest	OR (95% CI)^1^	OR (95% CI)^2^
Breakfast	1.65 (0.69-3.95)	1.55 (0.64-3.74)
Sugary drinks	1.14 (0.57-2.27)	1.15 (0.58-2.30)
Toothbrush	2.89 (1.33-6.25)	3.08 (1.41-6.76)
Nausea	2.68 (0.68-10.67)	2.82 (0.70-11.33)
Dizziness	3.33 (0.66-16.78)	2.88 (0.56-14.83)
Palpitations	2.80 (0.55-14.31)	2.78 (0.54-14.29)
School/preschool	3.56 (1.26-10.03)	3.68 (1.30-10.40)
Trusted Adults	5.14 (0.60-43.99)	5.26 (0.60-45.37)
Nagelkerke R^2^	19.3%	21.5%

^1^Mutually adjusted.

^2^Adjusted for sex.

When stratifying the multivariate regression by age, the analysis revealed that cases older than 8 years were almost four times more likely to brush their teeth irregularly or only once a day (OR 3.85; CI: 1.47–10.12) compared to controls. In contrast, there was no significant difference between cases and controls among the youngest children (OR: 1.17; CI: 0.32–4.26). This was also true for disliking school, where older children exposed to physical violence were almost four times more likely to dislike going to school (OR: 3.74; CI: 1.03–13.53) compared to controls (Table 7). These associations were not affected when adjusting for sex. No significant difference was observed among the 4- to 7-year-olds children (Table 7).

**Table 7:** Multivariate logistic regression of significant variables stratified by age.

Variable	Odds Ratio (95% CI)	
of interest	4-7 years^1,2^	8-14 years^1,2^
Breakfast	7.42 (0.84-65.65)	1.35 (0.53-3.48)
Sugary drinks	2.48 (0.88-6.96)	0.90 (0.39-2.10)
Toothbrush	1.17 (0.32-4.26)	3.85 (1.47-10.12)
Nausea	*NA*	2.04 (0.51-8.23)
Dizziness	*NA*	2.64 (0.52-13.43)
Palpitations	*NA*	4.05 (0.46-35.33)
School/preschool	4.73 (0.96-23.24)	3.74 (1.03-13.53)
Nagelkerke R^2^	16.7%	18.9%

^1^Mutually adjusted.

^2^Adjusted for sex.

*NA* = analyses not possible due to too few cases.

Given the question of whether the child received assistance with toothbrushing when they were 10 years or younger, we investigated this variable separately in a subpopulation of children aged 4 to 10 years. However, we found no statistically significant difference between controls and cases, even after adjusting for age and sex. When evaluating the overall number of concerns as a risk score (low risk = 0–2 concerns; medium risk = 3–5 concerns, and high risk = ≥ 6 concerns), cases reported significantly more concerns than controls (OR: 15.78; CI: 2.13–116.77). Stratified by age groups, children aged 8–14 years had higher odds of reporting having more than six concerns than controls (OR: 10.34; 1.36–78.59). None of the youngest children reported six or more concerns. For the 4-year-old to the 7-year-old, cases had 3.6 times higher odds of having 3–5 concerns compared to controls (OR: 3.53; 1.43–9.13) (Table 7). Among cases, girls reported significantly more concerns (≥ 6) than boys (OR: 2.38; 1.13–4.50). However, there were no significant differences in sex between groups when evaluating fewer than six concerns. The maximum number of concerns was 11 among cases and seven among controls.

## Discussion

Overall, children suspected of being exposed to physical violence reported more health-related concerns than controls. Nine of the 23 questions asked during the forensic examination were significantly associated with the outcome (Table 4), showing significant associations between physical child abuse and health concerns. Only toothbrushing and disliking school remained significant after adjustment in a multivariate analysis. Additionally, adjusting for sex slightly increased the number of significant predictors, and age stratification revealed only significant findings for children aged 8–14 years. In the multivariate logistic regression analysis stratified by age, the model achieved a maximum Nagelkerke R² of 0.189. This measure indicates that the model only explained 19% of the variability of the outcome (being exposed to physical violence) based on the questions asked during the examinations. A higher Nagelkerke R^2^ was found in the unstratified analyses after sex adjustment, indicating a slightly better model (Table 6). While these findings suggest that the variables included can provide some insight into the outcome, a significant portion of the variability remains unexplained. Lack of toothbrushing has been associated with child abuse in several studies [13,16,38,39], and could be an indicator of parental neglect, not meeting the need of the child to be reminded of or assisted with toothbrushing [40]. However, the severity of irregular toothbrushing was less pronounced in this study, as most of the children brushed their teeth once daily, rather than rarely, as mentioned in other studies.

School disengagement has also been linked to adverse childhood experiences, such as child abuse and behavioral problems with complex causes [12,41,42]. Previous studies have shown an association between child maltreatment and being disliked by peers, and experiencing more rejections by peers in school [43]. This could be a possible reason for the lack of enjoyment in school for some of the abused children in this study. Bullying has also been identified as an effective mediator for school disengagement [12]. In the current study, we did not have information on whether the children experienced being bullied. Previous research has examined the relationship between exposure to physical violence and possible psychosomatic symptoms in children [6,44,45]. Jernbro et al. found that children who had experienced physical violence reported significantly more psychosomatic symptoms (such as stomachache, headache, sleeplessness, dizziness, back pain, and loss of appetite) compared to their unexposed peers [6]. Similarly, Myhre et al. reported a higher prevalence of health complaints among exposed children using the CSSI-8 instrument, measuring the same complaints as Jernbro et al. Myhre et al. found that no specific symptom in the CSSI-8 was found to be more prominent than others, but there was a tendency for girls to report more symptoms [7]. Consistent with these findings, we also observed that children suspected of being exposed to violence reported more health concerns than unexposed children, with girls reporting more concerns than boys (cases only). This aligns with the findings of Hagborg et al., who found girls reported more internalizing symptoms, such as poorer mental well-being and increased psychosomatic symptoms, than boys in a population of 12- to 13-year-olds exposed to emotional violence [46]. However, these results must be interpreted cautiously due to the wide confidence intervals, indicating a lack of statistical power. Nonetheless, these findings may serve as supplementary information when evaluating potential child abuse cases, ultimately contributing to a reduction in underreported cases.

Missing data was more prevalent for cases than controls. One contributing factor was the lack of differentiation regarding which children were old enough to answer the specific questions, as all children were asked the same questions. If the child did not comprehend a question, it was left unanswered. However, the interviewing nurse adapted her technique to the child’s age. Additionally, the high proportion of missing data among cases resulted from revisions to the interview guide, as some variables were not included in the initial version. Identifying predictors of abuse-related poor health and well-being is complex and challenging. Numerous unknown confounders and mediators influence health, bodily distress, and well-being. It is well established that socioeconomic position can affect health and well-being. The controls in our study may have had different socioeconomic positions, but we did not have access to data regarding socioeconomic position and therefore, could not adjust for this confounder. Ethnicity, religion or origin has also been associated with corporal punishment and intimate partner violence [47–49], but these data were not available for both groups.

Disengagement in school is correlated with several lifestyle factors, including video gaming, skipping breakfast, daily snacking, feeling unwell upon waking, and being unsatisfied with one’s health [48]. However, we could not find an association between skipping breakfast and worrying sleep patterns, nor did we find statistical interaction effects between these variables. We observed that skipping breakfast was significantly associated with the outcome in the univariate analyses, as were sugary drinks (Table 4).

### Strengths and limitations

A strength of this study is the inclusion of police-reported cases of physical abuse, which in nature are presumed to have a high suspicion of physical abuse compared to the inclusion of a volunteer control group. However, the control group is small, making comparisons between groups a challenge. Also, we lacked information on socioeconomics in the study. Socioeconomics could be a confounder to the observed results if unequally distributed between the cases- and controls.

Another limitation of this study is that the interview guide was revised during the study period, although many questions remained unchanged across versions. Since some variables were introduced only in the revised version, these questions contributed to more missing data.

While individual variations exist within each age group, there are substantial developmental differences between a 4-year-old and a 14-year-old. Ideally, these children should be divided into age-specific groups and assessed using appropriate age instruments. However, this would require substantially larger sample size and preferably validated instruments. Some questions in the interviews were adapted from guidelines provided by the Danish Health Authorities for school nurses performing health interviews with children [30], and are, to our knowledge, not validated instruments. These questions were supplemented with the CSSI-8 instrument, inspired by its use at the Child Advocacy Center (CAC) in Oslo [7]. The CSSI-8 instrument includes a time aspect (how bothered the child has been within the last 14 days). Additionally, the CSSI-8 scale ranges from “Not bothered at all = 0”, “Bothered a little =1”, “Bothered some = 2”, “Bothered a lot = 3”, and “Bothered a whole lot = 4”. In this study, the time aspect was not included, and the frequency of the symptoms was adapted to fit the other questions in our questionnaire, e.g. bothered daily, weekly, monthly or rarely/never, making a 1:1 comparison difficult. In some of the variables, e.g. nausea, dizziness, palpitations, and fatigue, there were many missing answers. In our experience, it was harder to establish an understanding of these questions in the younger age groups as reflected in the age-stratified analyses (Table 5).

## Conclusion

Cases reported more health-related concerns than controls on several items. Among the 23 questions evaluated, nine were reported significantly more often by cases than by controls. This included health-related behavior, such as skipping breakfast (OR: 3.12; CI: 1.51–6.47), and a high intake of sugary drinks (OR: 2.01; CI: 1.15–3.54). Regarding other complaints, cases reported more bodily distress symptoms (5.21–8.95; CI: 1.54–38.38) and had fewer trusted adults (OR: 7.90; CI: 1.03–60.87).

In the adjusted and age-stratified multivariate model, two items remained significant. Cases between 8 and 14 years were almost four times more likely to brush their teeth once a day or less (OR: 3.85; CI: 1.47–10.12) and dislike school (OR: 3.74; CI: 1.03–13.53) compared to controls. A tendency to skip breakfast, high intake of sugary drinks, and dislike of school or preschool were also observed among the younger cases (<8 years), but these findings were not statistically significant. However, with the aim of identifying health concerns in the group at hand, the univariate analyses are suggested to be most relevant.

The questions used in the health-related interview were not found to be strong predictors of abuse, as only a maximum of 19% of the variation in being exposed to physical violence could be explained by the models. Nevertheless, the results underscore the importance of a preparedness of forensic personnel to initiate follow-up of health-related concerns, either by health professionals or by the social services. Future research should focus on further enhancing health-related questionnaires to be applied in a forensic setting to identify both risk groups of abuse and health-related concerns in such groups of children and to follow up on the results of interventions initiated based on such questionnaires.

## Supporting information

S1 File Full questionnaire used in the study to assess children’s health behaviors, symptoms, and well-being. (PDF)

S2 File Description of the variables and the wording used when asking children health-related questions during the forensic examination. (PDF)
