# Supplementary material for: Physical child abuse and self-reported health concerns: A case-control study including police-reported cases and unreported controls
Source: PLoS One. 2025 Sep 2;20(9):e0330601. doi: 10.1371/journal.pone.0330601 (PMC12404467; doi:10.1371/journal.pone.0330601)
Supplement: S1 File — (PDF) [file pone.0330601.s001.pdf]

ANAMNESIS	HEALTH INFORMATION
**Source of information**	Child examined ☐ Mother ☐ Father ☐ Teacher/Nursery staff ☐ Other:
**Diet**	Not relevant ☐
Breakfast	Every day ☐ Almost every day ☐ Irregularly ☐ Never ☐<br>Further clarification:
Lunch	Every day ☐ Almost every day ☐ Irregularly ☐ Never ☐<br>Further clarification:
Dinner	Every day ☐ Almost every day ☐ Irregularly ☐ Never ☐<br>Further clarification:
**Sweets**	Not relevant ☐
Consumption of sweets/chocolate	Daily ☐ 4-6 days/week ☐ 2-3 days/week ☐ 1 day/week ☐ Almost never/never ☐<br>Further clarification:
Consumption of sugary drinks	Daily ☐ 4-6 days/week ☐ 2-3 days/week ☐ 1 day/week ☐ Almost never/never ☐<br>Further clarification:<br>*See dietary guidelines in "Recommendations, sugar for children 3-6/7-15 years"*
**Dental hygiene**	Not relevant ☐<br>Teeth brushed twice a day ☐ Teeth brushed once a day☐ Further clarification:
Teeth brushing assisted by adult	Not relevant ☐ Yes ☐ No ☐ Further clarification:<br>*Relevant up to and including 10 years of age*
**Exercise pattern**	Less than 1 hour/day ☐ Approx. 1 hour/day ☐ More than 1 hour/day ☐<br>Further clarification:
Sports/hobbies	*Relevant to know whether the child's sports activities promote an increased heart rate, and if there is a health risk associated with the child's sport/hobby*
**Sleep pattern**	Approx. hours of sleep per night:<br>Feels generally alert and well rested ☐<br>Has difficulty falling asleep ☐<br>Often wakes up during the night ☐<br>Other/Further clarification:<br>*See the Danish Health Authority's guideline: Preventive healthcare services for children and adolescents, page 61*
**Urination pattern**	Able to stay dry in the daytime ☐ Able to stay dry at night ☐<br>Further clarification:
**Defecation pattern**	Able to control bowl movement, daytime ☐ Able to control bowl movement, night-time ☐<br>Experiences difficulties passing stools ☐ Experiences difficulties with runny stools ☐<br>Further clarification:
**Nausea**	Not relevant ☐ Rarely/never ☐ Monthly ☐ Weekly ☐ Daily/almost daily ☐<br>Further clarification:
**Dizziness**<br>**"and/or the feeling of passing out"**	Not relevant ☐ Rarely/never ☐ Monthly ☐ Weekly ☐ Daily/almost daily ☐<br>Further clarification:
**Palpitations**<br>**"Heart beating fast (when you are not exercising)"**	Not relevant ☐ Rarely/never ☐ Monthly ☐ Weekly ☐ Daily/almost daily ☐<br>Further clarification:
**Weakness**<br>**"Feeling weak in parts of the body"**	Not relevant ☐ Rarely/never ☐ Monthly ☐ Weekly ☐ Daily/almost daily ☐<br>Further clarification:

"Electronic versions of standards and checklists at the Department of Forensic Medicine, University of Copenhagen are the current version. Paper editions and supplements to publications may be out of date. This is for knowledge sharing only. If you are interested in using the documents for your own official work, please contact the Department of Forensic Medicine at the University of Copenhagen."

**Pain**	No ☐ Does not know ☐ Yes – ask where it hurts:
Headaches	Rarely/never ☐ Monthly ☐ Weekly ☐ Daily/almost daily ☐<br>Further clarification:
Stomach pain	Rarely/never ☐ Monthly ☐ Weekly ☐ Daily/almost daily ☐<br>Further clarification:
Back pain	Rarely/never ☐ Monthly ☐ Weekly ☐ Daily/almost daily ☐<br>Further clarification:
Pain in arms/legs	Rarely/never ☐ Monthly ☐ Weekly ☐ Daily/almost daily ☐<br>Further clarification:
**Happy at school**	Not relevant ☐<br>Happy at school: Yes ☐ No:<br>Further clarification:
**Social relationships with other children**	Not relevant ☐<br>Has friends: Yes ☐ No:<br>Further clarification:<br>*If the answer is no, ask how the child feels about not having any friends*
**Social relationships with adults**	Not relevant ☐<br>Has one/several adults to confide in: Yes ☐ No ☐<br>Further clarification:
**Known illnesses**	No ☐ Does not know ☐ Yes:<br>Further clarification:
**Asthma and allergies**	No ☐ Does not know ☐ Yes:<br>Further clarification:
**Medication**	No ☐ Further clarification:<br>Yes – Name of medicine, dose, and most recent ingestion:
**Information about pregnancy, birth, development (milestones)**	Not relevant ☐ No information ☐<br>Yes:
**Has followed the guidelines of the Danish childhood vaccination programme**	Yes ☐ Do not know ☐ No:
**Menarche**	Not relevant ☐ Not yet ☐ Yes – since when:<br>Further clarification:
**Other relevant health information**	None ☐ Further clarification:

"Electronic versions of standards and checklists at the Department of Forensic Medicine, University of Copenhagen are the current version. Paper editions and supplements to publications may be out of date. This is for knowledge sharing only. If you are interested in using the documents for your own official work, please contact the Department of Forensic Medicine at the University of Copenhagen."
