# Supplementary material for: Physical child abuse and self-reported health concerns: A case-control study including police-reported cases and unreported controls
Source: PLoS One. 2025 Sep 2;20(9):e0330601. doi: 10.1371/journal.pone.0330601 (PMC12404467; doi:10.1371/journal.pone.0330601)
Supplement: S2 File — (PDF) [file pone.0330601.s002.pdf]

Supplementary, S2.

Description of study variables

The health-related interviews were conducted in two versions. Between April 1 and December 16, 2020, 99 children were interviewed using version 1 of the health-related interview. From December 17, 2020, to December 31, 2023, a total of 253 children were interviewed using version 2, which was an updated and expanded version of the interview. Questions addressing somatic distress (nausea, dizziness, palpitations, fatigue, headache, abdominal pain, backpain, pain in arms and legs), additional health behaviors (exercise/physical activity), sugary drinks, and whether the child had friends, trusted adults and enjoyed school/preschool were first included in version 2 of the questionnaire.

1) **diet** (breakfast, lunch and dinner), **consumption of sweets and sugary drinks,** 2) **health behavior** (toothbrushing, exercise /physical activity), 3) **physical functions** (urination, defecation, sleep pattern), **4) somatic distress** (nausea, dizziness, palpitations, fatigue, headache, abdominal pain, back pain, pain in arms/legs, and 5) **predictors of thriving in school** (enjoying or disliking school, having friends and trusted adults).

1) **Diet and consumption of sweets** were evaluated through questions about the child's intake of breakfast, lunch, dinner, sweets, and sweetened beverages (e.g., soft drinks and squash). For example, the child was asked, *"Do you eat breakfast every day?"* If not, the child was asked to specify whether it was *almost every day, irregularly, or never*. The same questions were asked for lunch and dinner. For sweets and sweetened beverages, the child was asked, *"How often do you eat sweets or drink sweet drinks?"*. The nurse provided the following response options: *rarely/never, once a week, 2–3 times per week, 4–6 times per week, or daily.* If the child ate breakfast, lunch, and dinner rarely/never or irregularly, this was noted

as concerning behavior. Similarly, if the child consumed sweets and sugared drinks 4-6 times pr. weeks or daily, this was categorized as concerning behavior.

2) **Health behavior** was assessed in two domains: toothbrushing and physical activity. Toothbrushing was evaluated by asking the child, *"How often do you brush your teeth?"* An additional question for children aged 10 years and younger: *"Do you get help brushing your teeth?"* If a child responded negatively, clarification was noted in the text. If the child brushed their teeth once a day, irregularly, or never, this was categorized as concerning behavior. Physical activity was assessed by asking the child about their daily movements, including school play, leisure-time play, and cycling to and from school. The primary question was, *"Do you think you exercise more than 1 hour, less than 1 hour, or approximately 1 hour a day?"* To facilitate understanding, the nurse could begin by asking the child *"Do you know how long 1 hour is?"* For children who struggled with the concept of time, the nurse guided them in describing their daily activities and estimated their physical activity accordingly. If the child was less active than one hour a day, it was categorized as concerning behavior.

3) **Physical function** assessment included daytime wetting, defecation, and sleep patterns. The child was asked, *"Can you hold your pee, or do you have accidents? during the day and night? and during the night?"* Similarly, for bowel control, the child was asked, *"Can you hold your poop, or do you have accidents? during the day or night?* Additionally, questions concerning constipation and diarrhea were asked: *"Are you having trouble with constipation, meaning your poop being hard?" and "Do you have any trouble with diarrhea, meaning your poop is loose?".* Frequent daytime wetting was categorized as concerning for all ages. Nighttime wetting was categorized as concerning for children older than 5 years since enuresis first is defined at this age (Daley et al., 2025; Naiwen D Tu et. al,

2021). No child reported troubles with defecation during the day or at night. If the child reported frequent problems with constipation and/or diarrhea, this was categorized as concerning. Sleep patterns were assessed by asking children about their normal bedtime, wake-up time, and subjective feelings of restfulness. Additional questions about difficulties falling asleep and the frequency of waking at night. Sleep-related concerns were identified if the child reported two or more symptoms, for example, sleeping less than 8 hours per night, normal range for children according to (Hirshkowitz et al., 2015; The Danish Health Authority, 2024), or two of the following: experiencing frequent difficulties falling asleep, waking up during the night, and/or not having a feeling of restfulness in the morning. Isolated or occasional issues, such as experiencing these difficulties *sometimes* or *occasionally*, were not considered concerning.

4) **Somatic distress** was assessed using the revised version of the Children's Somatic Symptom Inventory (CSSI-8), an assessment tool measuring trauma symptom in children. This instrument is validated for children 8 years and older (Stone et al., 2024; Walker et al., 2009). The CSSI-8 includes eight non-specific somatic symptoms: headache, stomachache, lower back pain, pain in the arms or legs, nausea, dizziness, palpitations, and fatigue. In this study, the questions were adapted for all ages. To ensure comprehension, the nurses provided explanations for specific symptoms. For example, palpitations were described as follows: "*When you run or move, you can feel your heart beating fast, which is normal*". The child was then asked, *"Do you experience feeling your heart beating fast when resting or sitting still at school?"* If the response was positive, the child was asked to specify the frequency and whether it was *daily, weekly, monthly, or rarely/never. Daily and weekly* experiences were considered concerning. When the child affirmative responses to the questions, the nurse assessed whether the underlying reasons were cause of concern. For

instance, if the child experienced dizziness while spinning or nausea during car rides, these symptoms were not classified as concerning.

5) **Predictors of thriving and well-being in school/preschool** were assessed using three questions: *"Do you enjoy going to school/preschool?", 'Do you have friends?'* and *"Do you have trusted adults you can talk to?".* Responses indicating reasons for concern, such as *"I feel alone"* or *"I have no friends",* were categorized as concerning. Conversely, responses such as "*No, I do not enjoy school because it is boring", "No, I do not like waking up early",* and *"I have just moved and have no friends in the new area, but many in my old place"* were not considered concerning.

References

Daley, S. F., Gomez Rincon, M., & Leslie, S. W. (2025). Enuresis. I *StatPearls*. StatPearls Publishing. http://www.ncbi.nlm.nih.gov/books/NBK545181/

Hirshkowitz, M., Whiton, K., Albert, S. M., Alessi, C., Bruni, O., DonCarlos, L., Hazen, N., Herman, J., Adams Hillard, P. J., Katz, E. S., Kheirandish-Gozal, L., Neubauer, D. N., O'Donnell, A. E., Ohayon, M., Peever, J., Rawding, R., Sachdeva, R. C., Setters, B., Vitiello, M. V., & Ware, J. C. (2015). National Sleep Foundation's updated sleep duration recommendations: Final report. *Sleep Health*, *1*(4), 233–243. https://doi.org/10.1016/j.sleh.2015.10.004

Naiwen D Tu et. al. (2021). Nocturnal enuresis in children: Etiology and evaluation. https://www.uptodate.com/contents/nocturnal-enuresis-in-children-etiology-and-evaluation?topicRef=2863&source=see_link#H692664099

Stone, A. L., Garber, J., & Walker, L. S. (2024). The Children's Somatic Symptoms Inventory-8: Psychometric Properties of a Brief Measure of Somatic Distress. *Children*, *11*(11), 1326. https://doi.org/10.3390/children11111326

The Danish Health Authority. (2024). *Anbefalinger for søvnlængde – Til alle aldersgrupper, [Recommendations for Sleep Duration – For All Age Groups]*. https://www.sst.dk/-/media/Udgivelser/2024/Soevn/Anbefalinger-for-soevnlaengde.ashx

Walker, L. S., Beck, J. E., Garber, J., & Lambert, W. (2009). Children's Somatization Inventory: Psychometric Properties of the Revised Form (CSI-24). *Journal of Pediatric Psychology*, *34*(4), 430–440. https://doi.org/10.1093/jpepsy/jsn093
